# Effects of Inclisiran, Alirocumab, Evolocumab, and Evinacumab on Lipids: A Network Meta-Analysis

**DOI:** 10.31083/RCM25248

**Published:** 2025-02-22

**Authors:** Lin Zhang, Bin Li, Wei Chen, Wei Li, Huayun Yang, Diguang Pan

**Affiliations:** ^1^Department of Cardiology, Guilin People’s Hospital, 541002 Guilin, Guangxi, China

**Keywords:** total cholesterol, low density lipoprotein cholesterin, high density lipoprotein

## Abstract

**Background:**

Direct comparisons between the drugs are limited, and the dosing remains debatable. Therefore, the study aims to indirectly compare the efficacy and safety of inclisiran, alirocumab, evolocumab, and evinacumab in lipid-lowering through a network meta-analysis.

**Methods:**

Databases including PubMed, EMBASE, Web of Science, and the Cochrane Library were utilized to retrieve randomized controlled trials (RCTs). The search was conducted up to July 1, 2023. The Cochrane risk of bias tool was employed to appraise the quality of included studies. R software was used to conduct the Bayesian network meta-analysis.

**Results:**

Twenty-one RCTs with 10,835 patients were included. The network meta-analysis indicated that Evolocumab [mean difference (MD) = –60, 95% credibility interval (CrI) (–72, –49)] was the most effective (87%) in reducing low-density lipoprotein cholesterol (LDL-C), followed by alirocumab (71.4%) and inclisiran (47.2%), with placebo being the least effective (0.01%). In increasing high-density lipoprotein cholesterol (HDL-C), evolocumab [MD = 6.5, 95% CrI (3.2, 10)] ranked first (81.8%), followed by alirocumab (68.2%), with placebo again at the bottom (0.03%). In lowering total cholesterol, evolocumab [MD = –36, 95% CrI (–54, –19)] performed the best (86%), followed by alirocumab (64%), and placebo remained the least effective (0.04%). Regarding adverse events (AEs), evinacumab [odds ratio (OR) = 2, 95% CrI (1.17, 3.44)] ranked the highest (98.9%), followed by inclisiran (59.6%) and evolocumab (15.2%).

**Conclusions:**

Evolocumab appears to be the most effective in increasing HDL-C and reducing LDL-C and total cholesterol. Evinacumab shows the best safety profile with the lowest incidence of AEs.

**The PROSPERO registration:**

CRD42024570445, https://www.crd.york.ac.uk/prospero/display_record.php?RecordID=570445.

## 1. Introduction

Cardiovascular diseases (CVDs) are among the top causes of death globally [1]. Increased levels of low-density lipoprotein cholesterol (LDL-C) in the circulation are associated with the onset and advancement of coronary heart disease. LDL-C contributes to thrombosis formation and is a pivotal factor in the pathogenesis and progression of atherosclerosis. Additionally, there is a positive correlation between LDL-C levels and CVD-induced mortality [2, 3]. Clinical studies have demonstrated that reducing circulating LDL-C levels can lower the risk of significant cardiovascular events in patients with CVD [4, 5]. Therefore, controlling LDL-C levels to meet lipid targets in these patients is crucial, particularly in individuals at extreme risk of CVD. Statins are the cornerstone of lipid-lowering therapy for CVD, yet they are not always effective in treating patients at extreme cardiovascular risk [6]. Furthermore, a subset of CVD patients may experience statin-associated adverse reactions, including myalgia, central nervous system symptoms, liver function abnormalities, and diabetic symptoms. These reactions may necessitate statin dose reduction or a switch to alternative lipid-lowering therapies, impacting the lipid-lowering efficacy [7, 8]. With advancements in lipid-lowering treatments, novel agents such as proprotein convertase subtilisin/keexin type 9 (PCSK9) inhibitors have emerged. These agents have a different mechanism of action compared to statins and have shown significant lipid-lowering effects [9]. PCSK9 monoclonal antibodies, administered subcutaneously, reduce the likelihood of non-adherence associated with oral medications [10]. Recently, two novel lipid-modifying drugs, evolocumab and alirocumab, were approved by the United States Food and Drug Administration. Alirocumab is a monoclonal antibody developed to lower LDL-C. It functions by targeting and inhibiting PCSK9, a protein that binds to low-density lipoprotein (LDL) receptors (LDL-R) on liver cells. Evolocumab, a human monoclonal immunoglobulin G2 (IgG2), is effective in lowering LDL-C levels in patients with dyslipidemia. This binding facilitates the degradation of LDL-R, repressing the liver’s capacity to clear LDL-C from the blood [11]. These are fully human monoclonal antibodies targeting PCSK9, which can reduce the degradation of LDL-R by inhibiting PCSK9, thereby enhancing the metabolism of LDL-C. Serum LDL-C levels are increased after subcutaneous administration of evolocumab, with maximum plasma concentrations reached within 3 to 4 days and a half-life of 11 to 17 days [12]. Due to their distinct mechanisms of action, evolocumab, when combined with ezetimibe or statins, may have a synergistic effect in lowering LDL-C levels. Additionally, due to its high specificity, evolocumab is less likely to interact with other drugs [13, 14]. Angiopoietin-like 3 (ANGPTL3) is a liver-expressed secreted protein that can increase plasma levels of triglycerides (TG), LDL-C, and high-density lipoprotein cholesterol (HDL-C). Inhibiting ANGPTL3 can lower LDL-C levels independently of LDL-R function and may reduce the risk of cardiovascular events [15, 16]. As a chemically modified small interference RNA (siRNA), inclisiran inhibits the synthesis of the PCSK9 enzyme. Consequently, this upregulates LDL-R on hepatocytes, reducing plasma LDL-C concentrations [17].

The lipid-lowering efficacy and safety of inclisiran, alirocumab, evolocumab, and evinacumab remain contentious [18]. Direct comparisons between the drugs are inadequate, and the dosing remains debatable. This study is driven by the need to probe novel therapeutic strategies, with a particular focus on combining currently approved treatments with demoted drugs. By investigating the potential synergy between these treatments, we are committed to identifying more effective and efficient options for management.

## 2. Materials and Methods

The network meta-analysis was conducted following the PRISMA-NMA guidelines. The systematic review described herein was accepted by the online PROSPERO, the international prospective register of systematic reviews, affiliated with the National Institute for Health Research (CRD42024570445, https://www.crd.york.ac.uk/prospero/display_record.php?ID=CRD42024570445).

### 2.1 Literature Search

Cochrane, PubMed, Embase, and Web of Science databases were systematically searched for randomized controlled trials (RCTs) assessing the efficacy and safety of lipid-lowering therapies with inclisiran (Novartis AG, Basel, Switzerland), alirocumab (Regeneron Pharmaceuticals, Washington, DC, USA), evolocumab (Amgen Inc, Thousand Oaks, CA, USA), and evinacumab (Regeneron Pharmaceuticals, Washington, DC, USA), up until July 1, 2023. A set of medical subject headings (MeSH) and free-text terms relating to inclisiran, alirocumab, evolocumab, evinacumab, and hyperlipidemia was used. The** Supplementary Table 1** contains the full search strategy used.

### 2.2 Eligibility Criteria

Inclusion criteria: Our population consisted of adults with hyperlipidemia. The interventions investigated included inclisiran, alirocumab, evolocumab, or evinacumab, compared to a control group. Our outcomes of interest were LDL-C, HDL-C, and total cholesterol (primary outcomes), as well as adverse events (AEs) (secondary outcomes). Additionally, no restrictions were imposed on language or publication status.

Exclusion criteria: Duplicate studies, animal research, case reports, conference abstracts, reviews, articles with inaccessible comprehensive texts, and studies including patients with other organic diseases were excluded.

### 2.3 Data Extraction

The literature was screened and data were extracted by two independent reviewers. Potentially eligible studies were screened by downloading and reading titles, abstracts, and full texts of these studies. Discrepancies were resolved through discussion or by consulting a senior expert. Data extracted were cross-checked to ensure consistency and included the first author’s name, publication year, country of origin, sample size, participant demographics, treatment methods, and outcome measures.

### 2.4 Quality Assessment

The risk of bias was assessed following the latest recommendations of the Cochrane Handbook’s risk of bias assessment tool [19]. This tool encompasses five domains: bias from randomization, bias resulting from deviations of planned interventions, bias caused by incomplete outcome data, bias in outcome measurement, and bias in reported finding selection. Studies were categorized as having “minimal risk of bias”, “moderate concerns”, or “significant risk of bias”. Any disagreements between the two researchers were resolved by discourse or by involving a third party.

### 2.5 GRADE Assessment of Evidence Quality

The Grading of Recommendations Assessment, Development and Evaluation (GRADE) system was employed to evaluate the methodological quality of the evidence and ascertain the result quality [20]. Five factors that could downgrade the evidence quality were taken into account: study limitations, result inconsistencies, indirectness of evidence, imprecise or wide credibility intervals (CrIs), and bias of publication. In addition, another three factors were reviewed: the effect size, possible confounding factors, and dose-response relationship, which may also upgrade the quality of evidence. A detailed explanation of the evidence quality for each parameter is presented in Table 1.

**Table 1.** **GRADE assessment**.

Outcome	GRADE
LDL-C	Low
HDL-C	Moderat
Total cholesterol	Moderat
Adverse event	Low

GRADE, Grading of Recommendations Assessment, Development and Evaluation; LDL-C, low-density lipoprotein cholesterol; HDL-C, high-density lipoprotein cholesterol.

### 2.6 Data Analysis

Bayesian network meta-analysis was performed using a prior vague random effects model with the R 4.3.2 software (R Foundation for Statistical Computing, Vienna, Austria), and Markov Chain Monte Carlo method [21] was used to derive the optimal pooled estimate and probabilities of each therapeutic approach. Convergence of the model was evaluated using trace plots and Brooks-Gelman-Rubin plots. Continuous outcomes were expressed as posterior mean differences (MD) with their 95% CrI. The likelihood of each intervention being the most effective was estimated by computing the Surface Under the Cumulative Ranking curve (SUCRA) percentages. Network plots and funnel plots were generated using STATA 15.0 (StataCorp LLC, College Station, TX, USA). In the network plots, each node represented a medication, and the edges represented the available comparisons. The size of each node was proportional to the number of patients included. Cumulative probability plots were created using the ggplot2 package.

## 3. Results

### 3.1 Literature Screening Outcomes

**Fig. 1.**
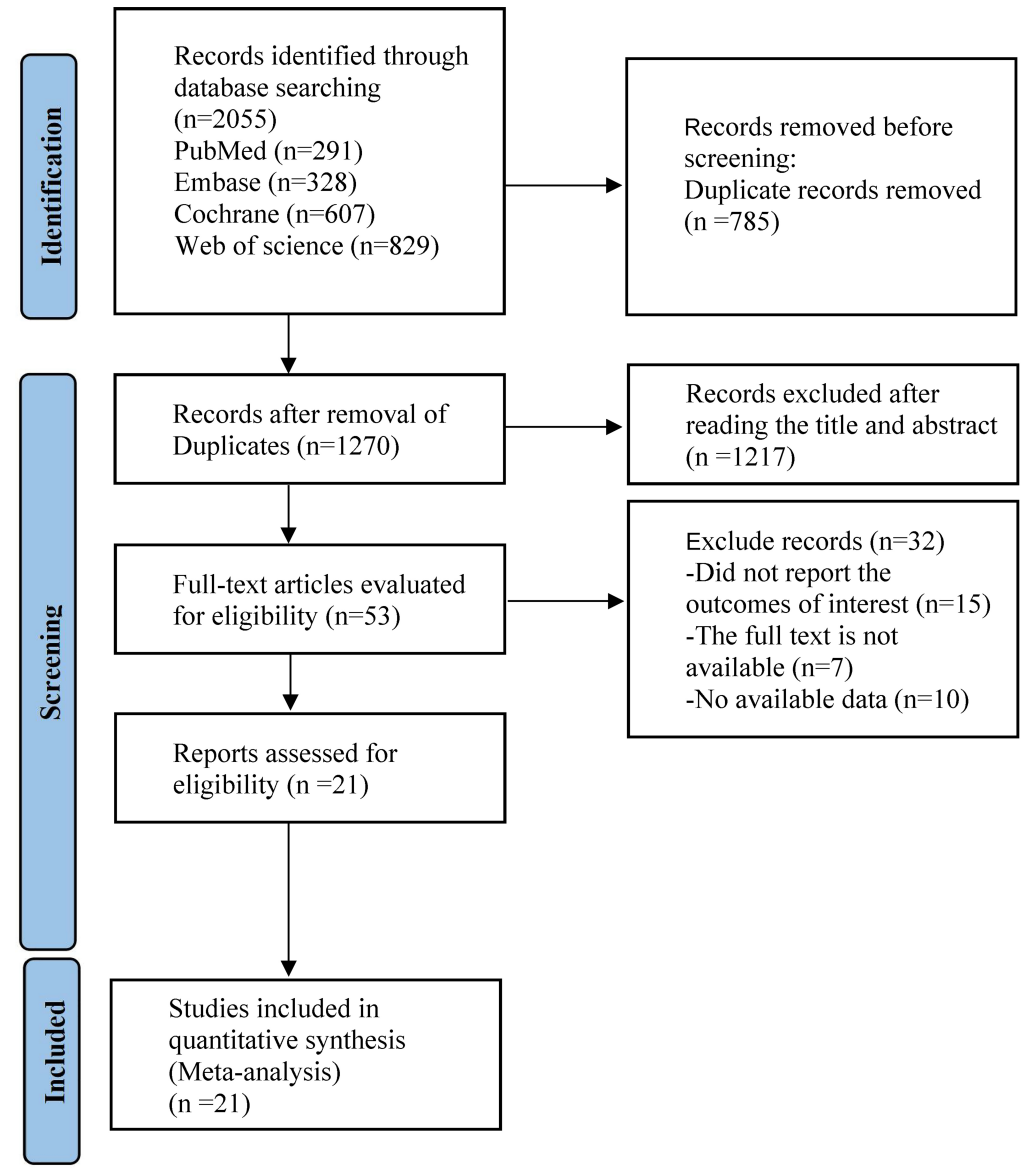
**PRISMA flow diagram of the study process**. PRISMA, Preferred Reporting Items for Systematic reviews and Meta-Analysis.

2055 articles were identified by searching databases. Following the removal of 785 duplicates, 1217 articles were excluded after screening titles and abstracts, and 32 more were excluded after full-text reading. Ultimately, 21 [22, 23, 24, 25, 26, 27, 28, 29, 30, 31, 32, 33, 34, 35, 36, 37, 38, 39, 40, 41, 42] articles were included in the analysis (Fig. 1).

### 3.2 Basic Characteristics and Quality Assessment of Included Studies

**Fig. 2.**
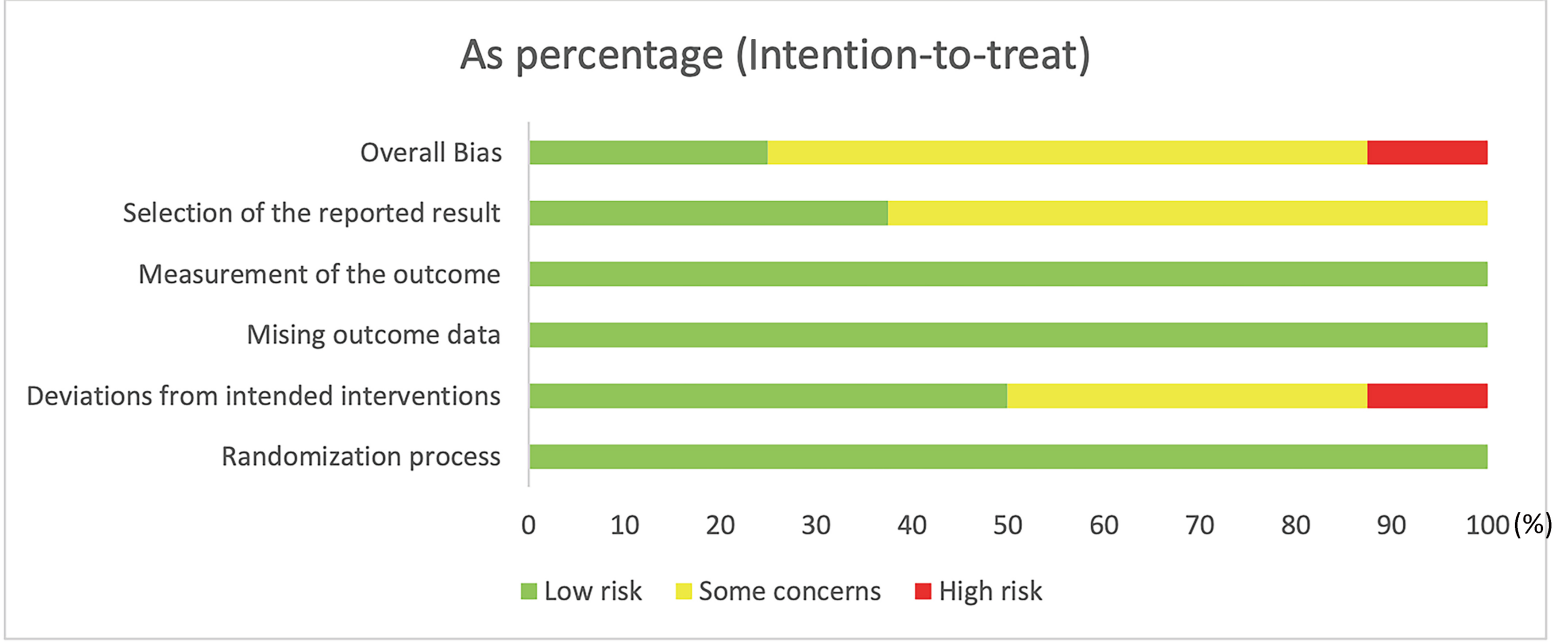
**Risk of bias summary**.

21 RCTs were included, comprising 10,835 patients. The interventions included inclisiran, alirocumab, evolocumab, and evinacumab. The attributes of the included studies are detailed in Table 2 (Ref. [22, 23, 24, 25, 26, 27, 28, 29, 30, 31, 32, 33, 34, 35, 36, 37, 38, 39, 40, 41, 42]). Blinding methods were explicitly explained across included studies. The main risk of bias was rooted in deviations from the intended interventions. The risk of bias assessment results are exhibited in Fig. 2.

**Table 2.** **Characteristics of the included studies**.

Study	Year	Country	Sample size		Gender (M/F)	Mean age (years)		Intervention		Outcome
			EG	CG		EG	CG	EG	CG	
Janik *et al*. [29]	2021	USA	1087	1084	1264/907	63	63	Alirocumab	Placebo	F1; F2; F3; F4
Blanco-Ruiz *et al*. [23]	2021	Spain	90	51	83/58	59.3	60	Alirocumab	Evolocumab	F1; F4
Santos *et al*. [39]	2020	Canada	104	53	69/88	13.7	13.7	Evolocumab	Placebo	F1; F4
Rosenson *et al*. [38]	2020	USA	121	39	60/100	54.5	52.4	Evinacumab	Placebo	F1; F4
Ray *et al*. [37]	2020	UK	781	780	1083/478	66.4	65.7	Inclisiran	Placebo	F4
			810	807	1587/37	64.8	64.8			
Raal *et al*. [35]	2020	Canada	43	22	35/30	44.3	36.7	Evinacumab	Placebo	F1; F4
Raal *et al*. [34]	2020	Canada	242	240	227/255	56	56	Inclisiran	Placebo	F1; F4
Boccara *et al*. [26]	2020	France	307	157	383/81	56.4	56.2	Evolocumab	Placebo	F4
Blom *et al*. [25]	2020	South Africa	45	24	34/35	42.3	45.4	Alirocumab	Placebo	F1; F2; F3; F4
Blom *et al*. [24]	2020	South Africa	657	324	419/562	61.3	61.3	Evolocumab	Placebo	F1
Teramoto *et al*. [40]	2019	Japan	107	56	103/60	62.6	64.6	Alirocumab	Placebo	F1; F2; F3; F4
Chao *et al*. [27]	2019	China	57	59	97/19	61.5	60	Alirocumab	Placebo	F1; F2; F3; F4
Ako *et al*. [22]	2019	Japan	93	89	144/38	61.8	60.5	Alirocumab	Placebo	F1; F2; F3; F4
Toth *et al*. [42]	2018	USA	336	283	296/323	56.4	57.1	Evolocumab	Placebo	F1; F2; F3
Koh *et al*. [32]	2018	Korea	97	102	164/35	61.2	60.1	Alirocumab	Placebo	F1; F2; F3; F4
Kastelein *et al*. [30]	2017	Norway	490	245	406/329	52.5	52.2	Alirocumab	Placebo	F1; F4
Teramoto *et al*. [41]	2016	Japan	144	72	131/85	60.3	61.8	Alirocumab	Placebo	F1; F2; F3; F4
Moriarty *et al*. [33]	2016	USA	41	21	36/26	59.5	57	Alirocumab	Placebo	F1; F4
Kiyosue *et al*. [31]	2016	Japan	202	202	244/160	62	61	Evolocumab	Placebo	F1; F2; F4
Ginsberg *et al*. [28]	2016	USA	72	35	57/50	49.8	52.1	Alirocumab	Placebo	F1; F2; F3; F4
Raal *et al*. [36]	2015	South Africa	110	54	95/69	52.6	51.1	Evolocumab	Placebo	F1; F2; F4

EG, experimental group; CG, control group; M, male; F, female; F1, LDL-C; F2, HDL-C; F3, total cholesterol; F4, adverse event.

### 3.3 LDL-C

**Fig. 3.**
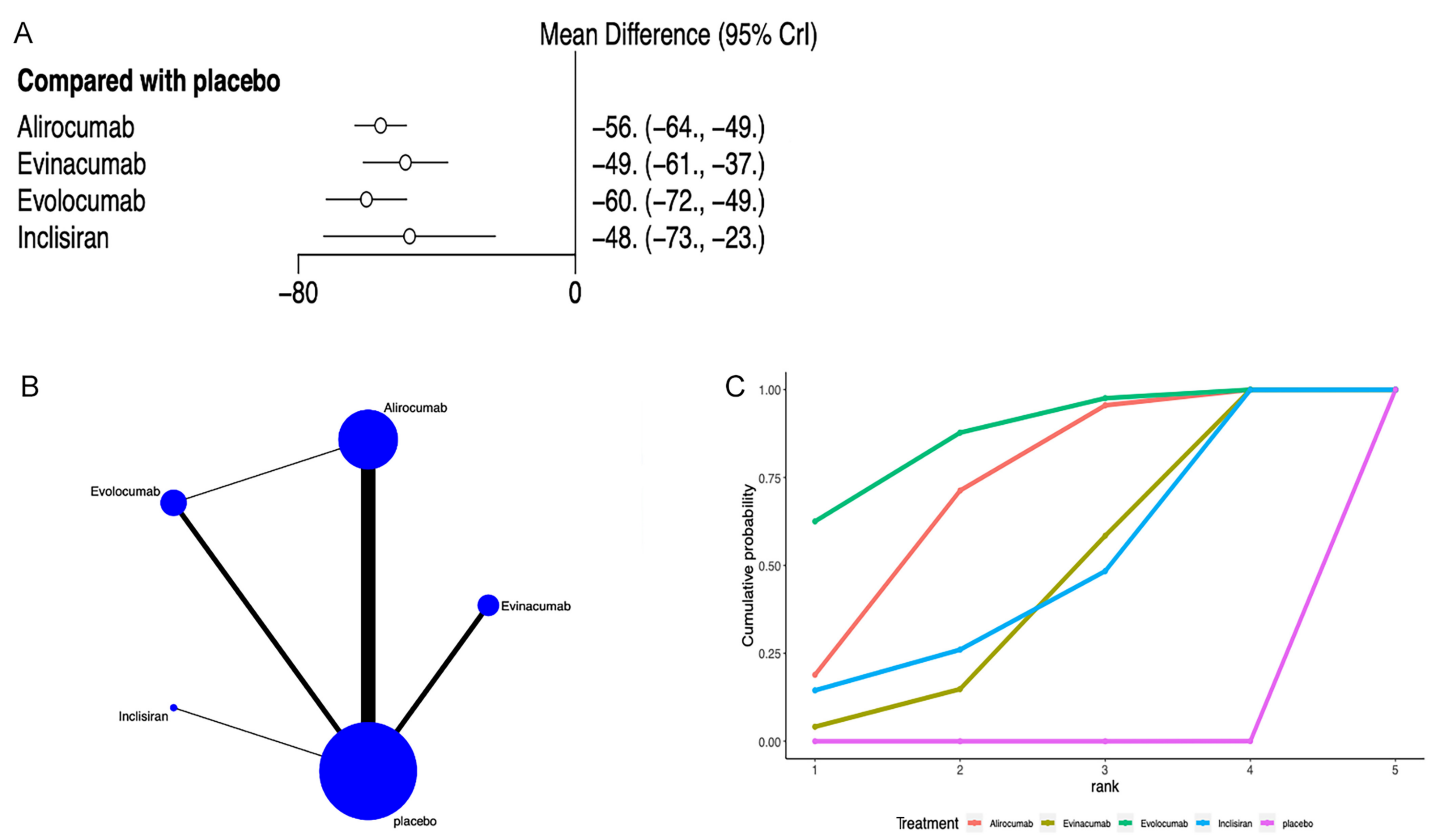
**Network meta-analysis of LDL-C**. (A) Network diagram; Network charts illustrating all the drug agents in the study. The line width is proportionate to the trial quantity comparing each treatment pair. The circle size is proportionate to the trial quantity involving the intervention. (B) Surface Under the Cumulative Ranking curve. (C) Forest map displaying the impact of drugs on LDL-C regulation. CrI, credibility interval; LDL-C, low-density lipoprotein cholesterol.

LDL-C levels were reported in 20 articles [22, 23, 24, 25, 26, 27, 28, 29, 30, 31, 32, 33, 34, 35, 36, 38, 39, 40, 41, 42] (Fig. 3). Network meta-analysis (Fig. 3B) demonstrated that compared to placebo, alirocumab [MD = –56, 95% CrI (–64, –49)], evinacumab [MD = –49, 95% CrI (–61, –37)], evolocumab [MD = –60, 95% CrI (–72, –49)], and inclisiran [MD = –48, 95% CrI (–73, –23)] significantly reduced LDL-C levels (Fig. 3A). The league table suggested no significant differences among inclisiran, alirocumab, evolocumab, and evinacumab (**Supplementary Table 2**). The SUCRA ranking unveiled that evolocumab was the best intervention in reducing LDL-C levels (87%), followed by alirocumab (71.4%) and inclisiran (47.2%), and placebo was the worst (0.01%) (Fig. 3C, Table 3).

**Table 3.** **SUCRA ranks**.

	LDL-C	HDL-C	Total cholesterol	Adverse event
Alirocumab	71.4%	68.2%	64.0%	26.2%
Evinacumab	44.3%	/	/	98.9%
Evolocumab	87.0%	81.8%	86.0%	15.2%
Inclisiran	47.2%	/	/	59.6%
Placebo	0.01%	0.03%	0.04%	50.0%

LDL-C, low-density lipoprotein cholesterol; HDL-C, high-density lipoprotein cholesterol; SUCRA, Surface Under the Cumulative Ranking curve.

### 3.4 HDL-C

**Fig. 4.**
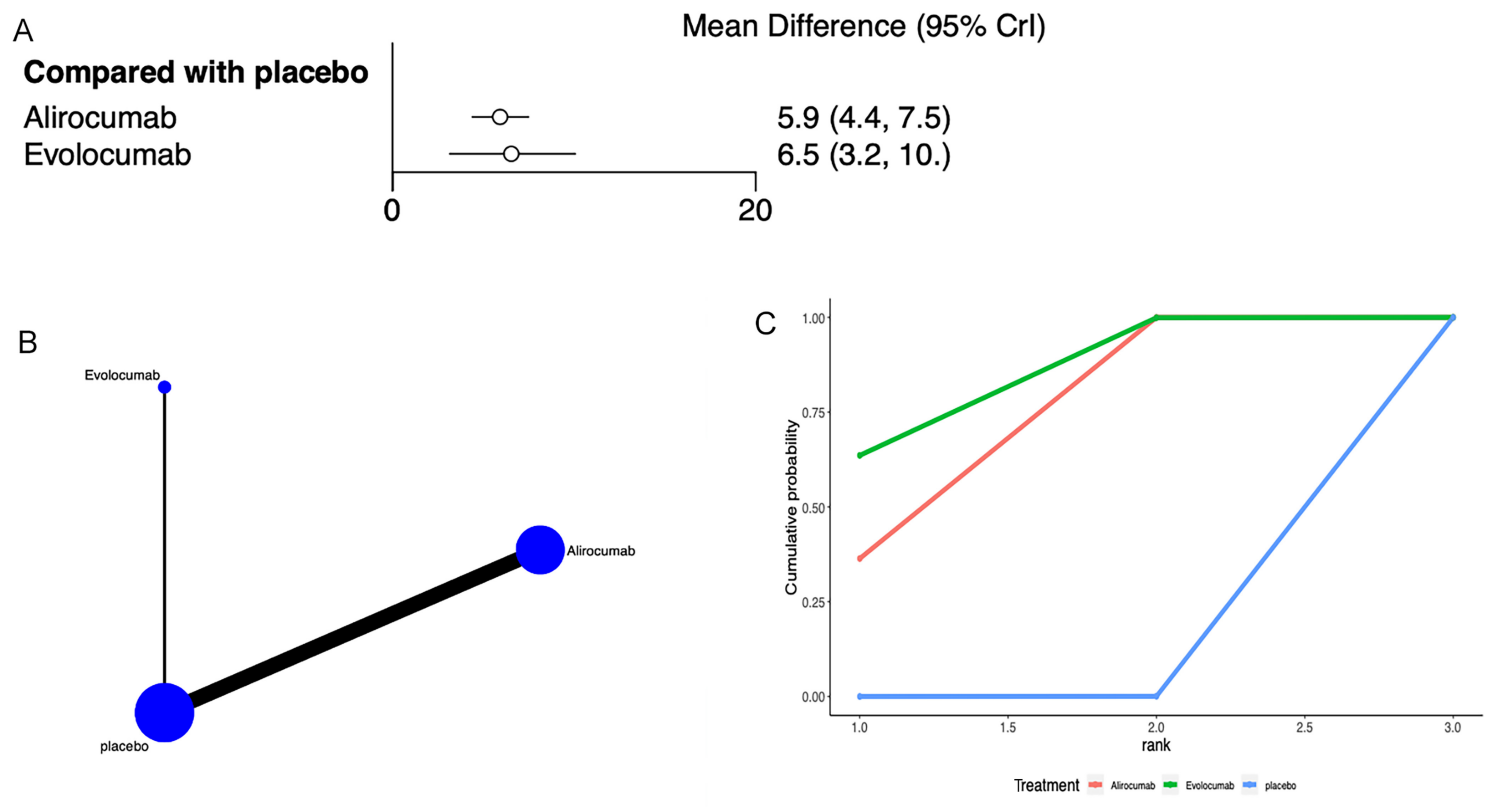
**Network meta-analysis of HDL-C**. (A) Network diagram; Network charts illustrating all the drug agents in the study. The line width is proportionate to the trial quantity comparing each treatment pair. The circle size is proportionate to the trial quantity involving the intervention. (B) Surface Under the Cumulative Ranking curve. (C) Forest map displaying the impact of drugs on LDL-C regulation. CrI, credibility interval; HDL-C, high-density lipoprotein cholesterol.

HDL-C levels were reported in 11 articles [22, 24, 27, 28, 29, 31, 32, 36, 40, 41, 42] (Fig. 4). Network meta-analysis (Fig. 4B) showed that compared to placebo, alirocumab [MD = 5.9, 95% CrI (4.4, 7.5)], and evolocumab [MD = 6.5, 95% CrI (3.2, 10)] both increased HDL-C levels (Fig. 4A). The league table indicated no significant differences between alirocumab and evolocumab (**Supplementary Table 3**). The SUCRA ranking revealed that evolocumab was the most effective in increasing HDL-C levels (81.8%), followed by alirocumab (68.2%), and placebo ranked last (0.03%) (Fig. 4C, Table 3).

### 3.5 Total Cholesterol

**Fig. 5.**
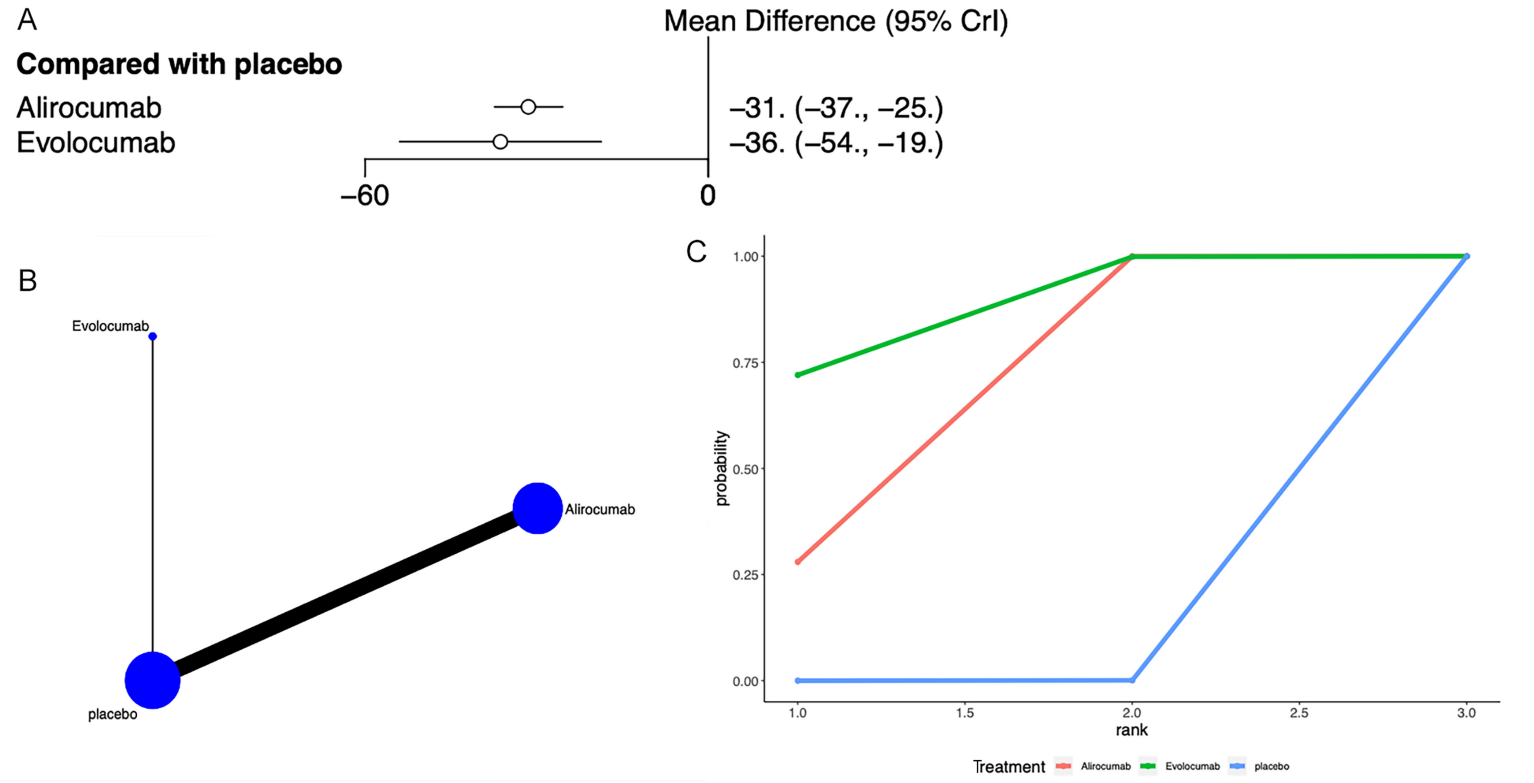
**Network meta-analysis of total cholesterol**. (A) Network diagram; Network charts illustrating all the drug agents in the study. The line width is proportionate to the trial quantity comparing each treatment pair. The circle size is proportionate to the trial quantity involving the intervention. (B) Surface Under the Cumulative Ranking curve. (C) Forest map displaying the impact of drugs on LDL-C regulation. CrI, credibility interval; LDL-C, low-density lipoprotein cholesterol.

Total cholesterol was addressed in 9 articles [22, 24, 27, 28, 29, 32, 40, 41, 42] (Fig. 5). Network meta-analysis (Fig. 5B) found that compared to placebo, alirocumab [MD = –31, 95% CrI (–37, –25)], and evolocumab [MD = –36, 95% CrI (–54, –19)] both reduced total cholesterol levels (Fig. 5A). The league table indicated no significant differences between alirocumab and evolocumab (**Supplementary Table 4**). The SUCRA ranking showed that evolocumab ranked first (86%), followed by alirocumab (64%), and placebo ranked last (0.04%) (Fig. 5C, Table 3).

### 3.6 Adverse Events

**Fig. 6.**
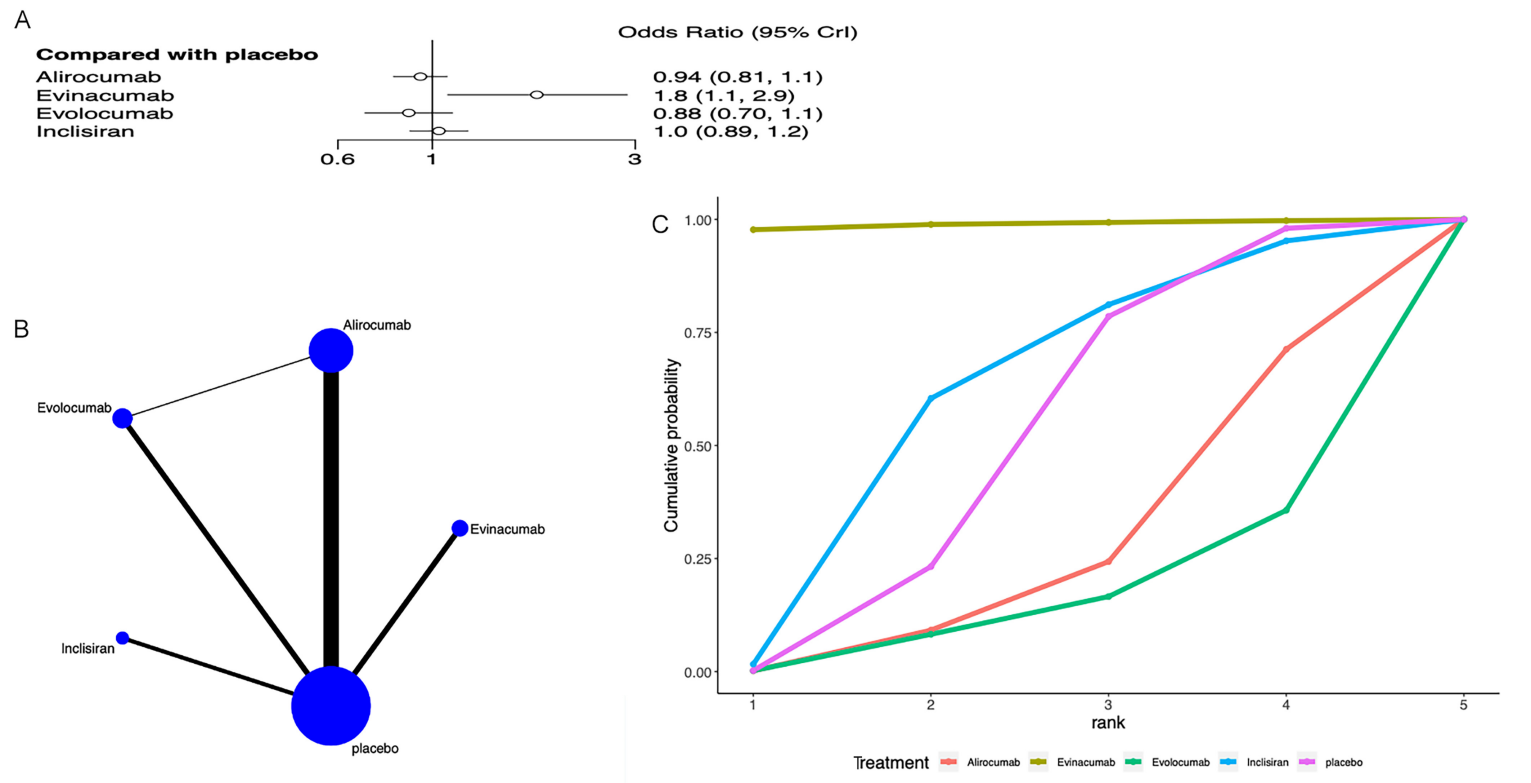
**Network meta-analysis of adverse events**. (A) Network diagram; Network charts illustrating all the drug agents in the study. The line width is proportionate to the trial quantity comparing each treatment pair. The circle size is proportionate to the trial quantity involving the intervention. (B) Surface Under the Cumulative Ranking curve. (C) Forest map displaying the impact of drugs on LDL-C regulation. CrI, credibility interval; LDL-C, low-density lipoprotein cholesterol.

AEs were discussed in all 21 studies [22, 23, 24, 25, 26, 27, 28, 29, 30, 31, 32, 33, 34, 35, 36, 37, 38, 39, 40, 41, 42]. All adverse events are presented in Fig. 6. The network meta-analysis formed a closed loop (Fig. 6B), so we performed local inconsistency tests. The results suggested no differences between the direct, indirect, and network meta-analysis comparisons for evolocumab vs alirocumab, placebo vs alirocumab, and placebo vs evolocumab (**Supplementary Fig. 1**). Compared to placebo, evinacumab [OR = 1.8, 95% CrI (0.71, 1.1)] increased the incidence of AEs (Fig. 6A). The league table showed that the incidence of AEs was lower with evinacumab compared to evolocumab [OR = 2, 95% CrI (1.17, 3.44)] and inclisiran [OR = 1.7, 95% CrI (1.02, 2.84)] (**Supplementary Table 5**). The SUCRA ranking revealed that evinacumab ranked first (98.9%), followed by inclisiran (59.6%) and then evolocumab (15.2%) (Fig. 6C, Table 3).

### 3.7 Publication Bias

The publication bias for LDL-C, HDL-C, total cholesterol, and AEs was assessed with funnel plots, suggesting a low likelihood of publication bias for these outcomes (**Supplementary Figs. 2–5**).

## 4. Discussion

This study represents the first network meta-analysis to evaluate the efficacy and safety of lipid-lowering treatments with inclisiran, alirocumab, evolocumab, and evinacumab. The findings suggest that in the reduction of LDL-C and total cholesterol, as well as in the elevation of HDL-C, evolocumab is the most effective, followed by alirocumab. Alirocumab is a monoclonal antibody targeting PCSK9, a serine protease synthesized in the liver and secreted into the plasma, where it exerts its function by binding to LDL-Rs. Normally, LDL-Rs are primarily found on the surface of hepatocytes. They bind to LDL-C particles to form complexes, which are then endocytosed into hepatocytes, where the LDL-C particles are degraded in lysosomes, allowing the LDL-R to recycle back to the hepatocyte surface. Inhibiting the degradation of cholesterol receptors thus leads to lower cholesterol levels in the blood [43, 44]. McKenney *et al*. [45] included 182 participants treated with subcutaneous alirocumab, reporting a significant reduction in LDL-C levels after 12 weeks. Dias *et al*. [46] first evaluated the effects and safety of evolocumab in patients with hypercholesterolemia, healthy volunteers, and individuals with heterozygous familial hypercholesterolemia; the results showed that evolocumab markedly reduced their serum LDL-C levels. It has been reported that evolocumab can safely and effectively reduce LDL-C levels by more than 20% in patients with familial hypercholesterolemia. By binding to PCSK9, evolocumab inhibits PCSK9’s interaction with LDL-R, thereby elevating the number of LDL-R on the liver surface. This enhancement in LDL-R availability boosts the liver’s capacity to remove LDL-C from the blood, contributing to a significant reduction in blood cholesterol levels and a decreased risk of cardiovascular events [47]. Regarding dosage, the administration of either 140 mg biweekly or 420 mg monthly was demonstrated to be safe and effective in improving LDL-C levels [36]. Evolocumab also proved most effective in raising HDL-C levels, followed by alirocumab. Dyslipidemia is well-known as a risk factor for atherosclerotic cardiovascular disease (ASCVD), and LDL-C is closely linked to the occurrence and progression of CVD. HDL not only induces reverse cholesterol transport from the periphery to the liver but also prevents LDL-C oxidation, shielding endothelial cells from oxidative damage. Furthermore, HDL enhances the production of nitric oxide by endothelial cells, exerting anti-apoptotic and anti-inflammatory effects, thereby inhibiting the formation of atherosclerosis [48, 49]. Stein *et al*. [50] summarized data from 1791 patients treated with evolocumab, finding that while it effectively lowered triglyceride levels in patients with hypertriglyceridemia, the reduction in LDL-C was not superior to that achieved with statins. Additional research utilized evolocumab to lower lipids in severe hypertriglyceridemia (HTG) patients and performed whole-exome sequencing to identify potential genetic causes of dyslipidemia. The findings suggested that PCSK9 inhibitors significantly reduce triglyceride levels regardless of the presence of mutations. In terms of adverse drug reactions, evinacumab had the lowest incidence of AEs, followed by inclisiran, with evolocumab having the highest. In a phase II clinical trial [51], 9 patients experienced at least one AE, such as nasopharyngitis, flu-like symptoms, dizziness, rhinorrhea, nausea, limb pain, and weakness, yet none discontinued treatment due to these side effects. The most common adverse reactions related to inclisiran are myalgia, headache, fatigue, nasopharyngitis, back pain, hypertension, diarrhea, and dizziness. Other adverse reactions in observational reports include no increase in C-reactive protein, tumor necrosis factor-α, and interleukin-6 following prolonged inclisiran treatment, suggesting that PCSK9 does not partake in systemic inflammatory processes. This supports that inclisiran is unlikely to adversely affect the immune system in patients at high risk for CVD. This aligns with clinical trial findings for the anti-evinacumab monoclonal antibody. Potential disadvantages of inclisiran are possible adverse effects that might be noticed after several years of treatment, as inclisiran remains active for six months, making it challenging to reverse any potential long-term adverse effects [52]. However, PCSK9 inhibitors are typically not used as a monotherapy. They are most effective when combined with statins or ezetimibe. Statins function by inhibiting cholesterol synthesis in the liver, while ezetimibe reduces cholesterol absorption from the diet. Combining a PCSK9 inhibitor with these therapies can achieve a more substantial reduction in LDL-C levels compared to either treatment alone. This synergistic approach facilitates optimal cholesterol control, especially in patients who do not meet their LDL-C targets with statins alone or who are intolerant to statins [53, 54].

Although this study confirms the benefits of evolocumab in improving HDL-C, and reducing LDL-C and total cholesterol, several limitations should be considered. Firstly, the quantity of included studies was limited, and direct comparisons between studies were rare, contributing to significant heterogeneity that may affect the credibility of the outcomes. Secondly, in different studies, we could not determine which statins and doses were received before and during the initiation of new lipid-lowering drug therapy; whether all patients received the maximum tolerated dose of statins, some studies did not specify the statins used, and some studies used some or no other statins. Thirdly, the population we included in our hyperlipidemia study, including these (familial hypercholesterolemia [FH] heterozygotes), FH homozygotes, FH compound heterozygotes, or patients who did not reach target LDL cholesterol values after acute coronary syndromes, was also a limitation of our study.

## 5. Conclusions

The present study reveals that evolocumab may offer the most pronounced benefits in elevating HDL-C, as well as reducing LDL-C and total cholesterol levels. Furthermore, evinacumab exhibited the most favorable safety profile with the lowest incidence of adverse drug reactions. However, due to substantial heterogeneity among the studies, additional high-quality, multicenter, RCTs are required to corroborate our conclusions.

## Availability of Data and Materials

The datasets used and/or analyzed during the current study are available from the corresponding author on reasonable request.

## Supplementary Material

Supplementary material associated with this article can be found, in the online version, at https://doi.org/10.31083/RCM25248.
