# Supplementary material for: Effects of Inclisiran, Alirocumab, Evolocumab, and Evinacumab on Lipids: A Network Meta-Analysis
Source: Rev Cardiovasc Med. 2025 Feb 22;26(2):25248. doi: 10.31083/RCM25248 (PMC11868915; doi:10.31083/RCM25248)
Supplement: Supplementary file 1 [file 2153-8174-26-2-25248-s1.zip › Supplementary Material.docx]

**Supplementary materials**

[Supplementary Table 1 Search strategy 2](#_Toc15534)

[Supplementary Table 2 LDL-C league table 4](#_Toc17886)

[Supplementary Table 3 HDL-C league table 4](#_Toc19673)

[Supplementary Table 4 Total cholesterol league table 4](#_Toc28147)

[Supplementary Table 5 Adverse events league table 4](#_Toc20335)

[Supplemental Fig. 1 Local inconsistency test 5](#_Toc26576)

[Supplemental Fig. 2 Egger test of LDL-C 5](#_Toc1695)

[Supplemental Fig. 3 Egger test of HDL-C 6](#_Toc22284)

[Supplemental Fig. 4 Egger test of total cholesterol 6](#_Toc28963)

[Supplemental Fig. 5 Egger test of adverse event 7](#_Toc2809)

**Supplementary Table 1** Search strategy

**PUBMED**

(randomized controlled trial[Publication Type] OR randomized[Title/Abstract] OR placebo[Title/Abstract]) AND (((((("ALN-PCS" [Supplementary Concept]) OR ("evinacumab" [Supplementary Concept])) OR ("evolocumab" [Supplementary Concept])) OR ("alirocumab" [Supplementary Concept])) OR ((((((((((((((((((((ALN-PCS[Title/Abstract]) OR (ALN-PCSsc[Title/Abstract])) OR (inclisiran[Title/Abstract])) OR (evinacumab[Title/Abstract])) OR (evinacumab-dgnb[Title/Abstract])) OR (evkeeza[Title/Abstract])) OR (REGN-1500[Title/Abstract])) OR (REGN1500[Title/Abstract])) OR (evolocumab[Title/Abstract])) OR (repatha[Title/Abstract])) OR (AMG-145[Title/Abstract])) OR (AMG 145[Title/Abstract])) OR (alirocumab[Title/Abstract])) OR (SAR236553[Title/Abstract])) OR (SAR-236553[Title/Abstract])) OR (REGN-727[Title/Abstract])) OR (monoclonal antibody REGN727[Title/Abstract])) OR (REGN727 monoclonal antibody[Title/Abstract])) OR (REGN727[Title/Abstract])) OR (praluent[Title/Abstract]))) AND (("Hypercholesterolemia"[Mesh]) OR ((((((((((((((Hypercholesterolemia[Title/Abstract]) OR (Hypercholesterolemias[Title/Abstract])) OR (High Cholesterol Levels[Title/Abstract])) OR (Cholesterol Level, High[Title/Abstract])) OR (Cholesterol Levels, High[Title/Abstract])) OR (High Cholesterol Level[Title/Abstract])) OR (Level, High Cholesterol[Title/Abstract])) OR (Levels, High Cholesterol[Title/Abstract])) OR (Elevated Cholesterol[Title/Abstract])) OR (Cholesterol, Elevated[Title/Abstract])) OR (Cholesterols, Elevated[Title/Abstract])) OR (Elevated Cholesterols[Title/Abstract])) OR (Hypercholesteremia[Title/Abstract])) OR (Hypercholesteremias[Title/Abstract]))))

**Cochrane library**

ID Search Hits

#1 MeSH descriptor: [Hypercholesterolemia] explode all trees 3620

#2 (Hypercholesterolemia):ti,ab,kw OR (Hypercholesterolemias):ti,ab,kw OR (High Cholesterol Levels):ti,ab,kw OR (Cholesterol Level, High):ti,ab,kw OR (Cholesterol Levels, High):ti,ab,kw 20188

#3 (High Cholesterol Level):ti,ab,kw OR (Level, High Cholesterol):ti,ab,kw OR (Levels, High Cholesterol):ti,ab,kw OR (Elevated Cholesterol):ti,ab,kw OR (Cholesterol, Elevated):ti,ab,kw 15907

#4 (Cholesterols, Elevated):ti,ab,kw OR (Elevated Cholesterols):ti,ab,kw OR (Hypercholesteremia):ti,ab,kw OR (Hypercholesteremias):ti,ab,kw 77

#5 #1 OR #2 OR #3 OR #4 21587

#6 (ALN-PCS):ti,ab,kw OR (ALN-PCSsc):ti,ab,kw OR (inclisiran):ti,ab,kw OR (evinacumab):ti,ab,kw OR (evinacumab-dgnb):ti,ab,kw 114

#7 (evkeeza):ti,ab,kw OR (REGN-1500):ti,ab,kw OR (REGN1500):ti,ab,kw OR (evolocumab):ti,ab,kw OR (repatha):ti,ab,kw 444

#8 (AMG-145):ti,ab,kw OR (AMG 145):ti,ab,kw OR (alirocumab):ti,ab,kw OR (SAR236553):ti,ab,kw OR (SAR-236553):ti,ab,kw 526

#9 (REGN-727):ti,ab,kw OR (monoclonal antibody REGN727):ti,ab,kw OR (REGN727 monoclonal antibody):ti,ab,kw OR (REGN727):ti,ab,kw OR (praluent):ti,ab,kw 69

#10 #6 OR #7 OR #8 OR #9 976

#11 #5 AND #10 607

**Supplementary Table 2** LDL-C league table

| MD 95%CI | | | | |
| --- | --- | --- | --- | --- |
| Alirocumab |  |  |  |  |
| -7.16 (-21.47, 7.02) | Evinacumab |  |  |  |
| 4.11 (-9.12, 17.37) | 11.31 (-5.62, 28.09) | Evolocumab |  |  |
| -8.37 (-34.21, 17.63) | -1.19 (-28.68, 26.53) | -12.51 (-39.81, 14.91) | Inclisiran |  |
| -56.25 (-63.67, -48.82)* | -49.07 (-61.27, -36.9)* | -60.38 (-71.97, -48.74)* | -47.89 (-72.67, -23.16)* | placbeo |

* means P<0.05

**Supplementary Table 3** HDL-C league table

| MD 95%CI | | |
| --- | --- | --- |
| Alirocumab |  |  |
| -0.61 (-4.45, 3.09) | Evolocumab |  |
| 5.92 (4.4, 7.48)* | 6.54 (3.16, 10.06)* | placbeo |

* means P<0.065

**Supplementary Table 4** Total cholesterol league table

| MD 95%CI | | |
| --- | --- | --- |
| Alirocumab |  |  |
| 4.88 (-13.62, 23.55) | Evolocumab |  |
| -31.45 (-37.33, -25.45)* | -36.33 (-53.94, -18.73)* | placbeo |

* means P<0.05

**Supplementary Table 5** Adverse events league table

| OR 95%CI | | | | |
| --- | --- | --- | --- | --- |
| Alirocumab |  |  |  |  |
| 0.53 (0.32, 0.88)* | Evinacumab |  |  |  |
| 1.06 (0.81, 1.4) | 2 (1.17, 3.44)* | Evolocumab |  |  |
| 0.9 (0.73, 1.12) | 1.7 (1.02, 2.84)* | 0.85 (0.64, 1.13) | Inclisiran |  |
| 0.94 (0.81, 1.08) | 1.76 (1.09, 2.87)* | 0.88 (0.7, 1.11) | 1.04 (0.89, 1.21) | placbeo |

* means P<0.05


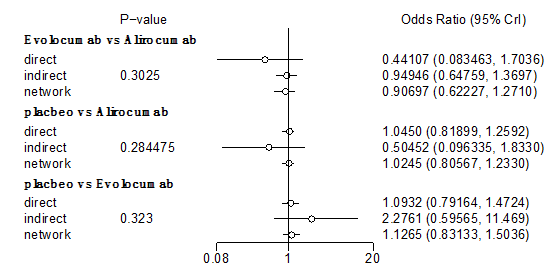


**Supplemental Fig. 1** Local inconsistency test


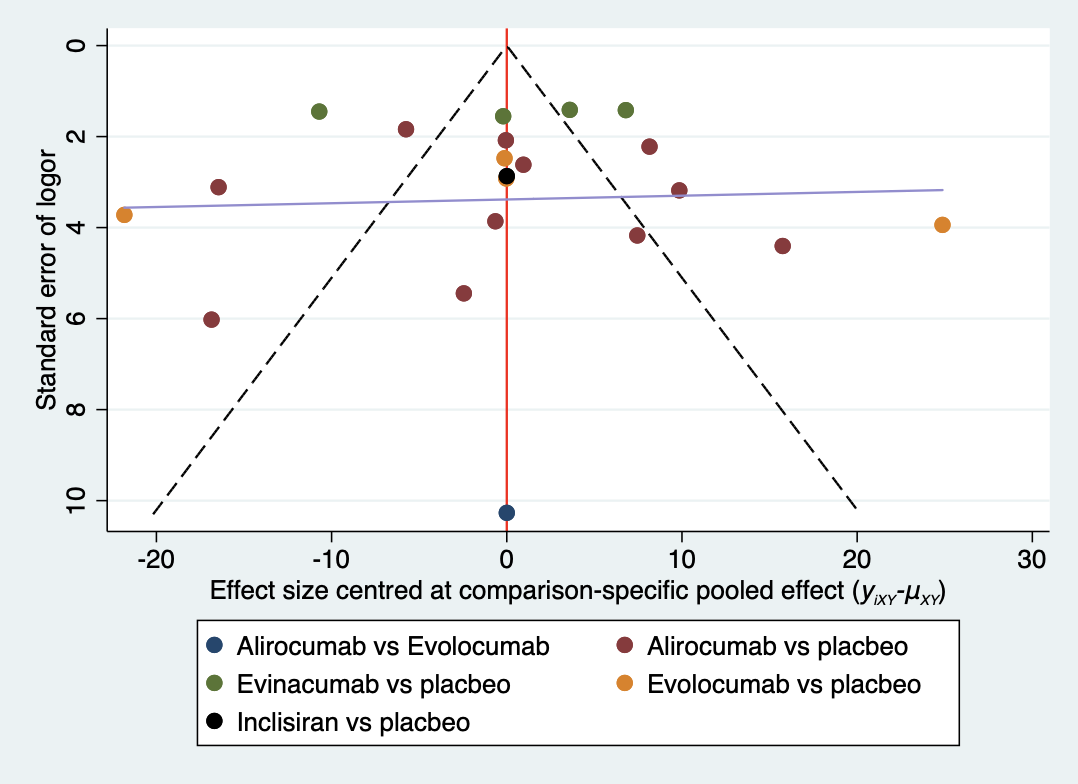


**Supplemental Fig. 2** Egger test of LDL-C


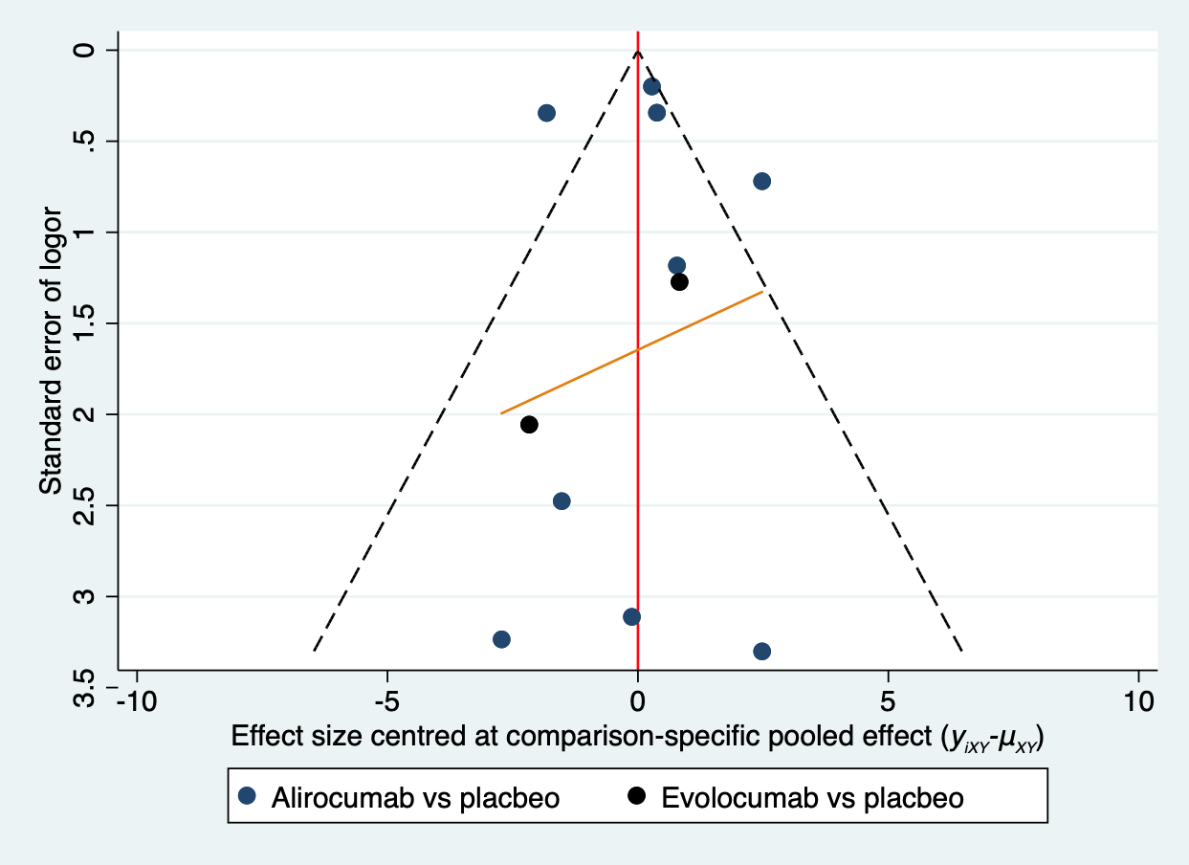


**Supplemental Fig. 3** Egger test of HDL-C


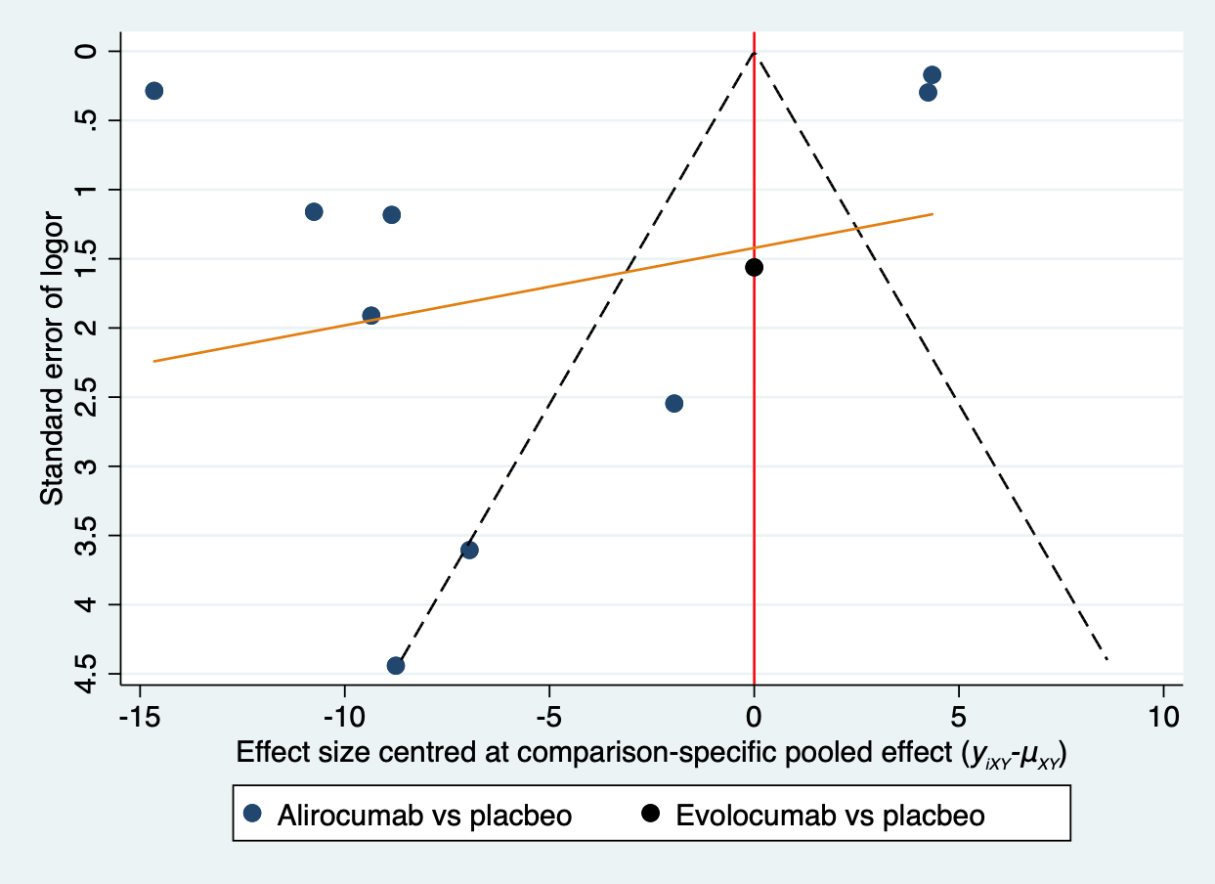


**Supplemental Fig. 4** Egger test of total cholesterol


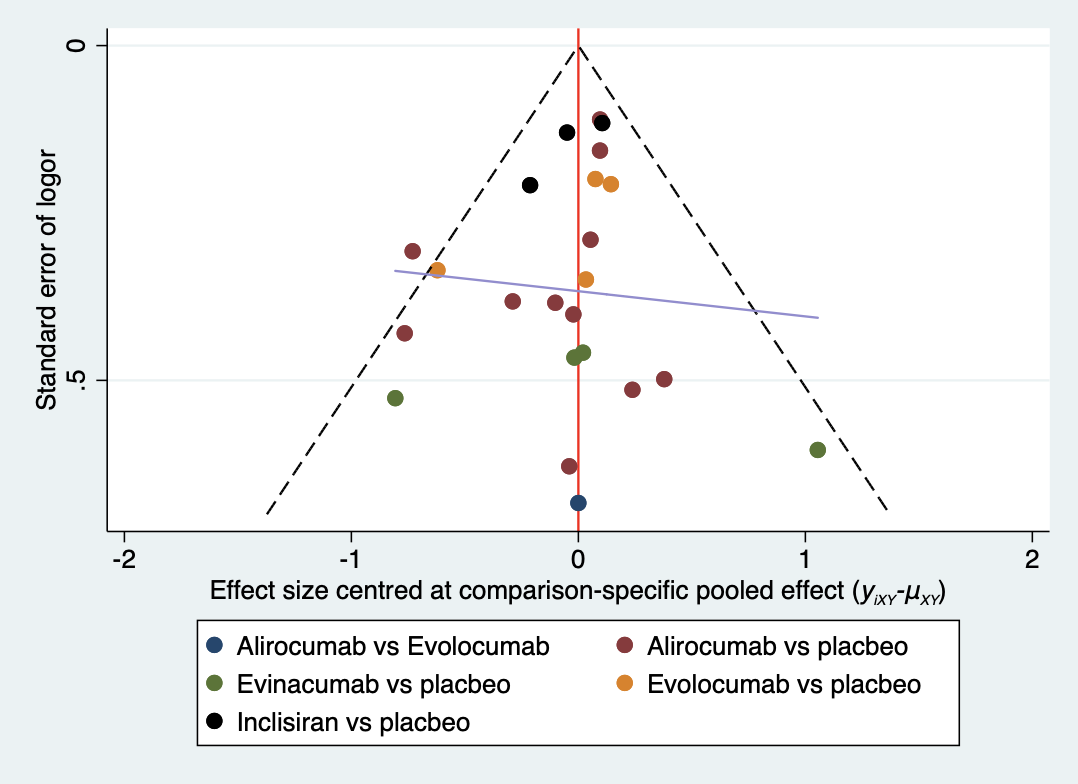


**Supplemental Fig. 5** Egger test of adverse event
